# Supplementary material for: WordCommentsAnalyzer: A windows software tool for qualitative research
Source: F1000Res. 2018 Sep 4;7:536. Originally published 2018 May 3. [Version 2] doi: 10.12688/f1000research.14819.2 (PMC6137411; doi:10.12688/f1000research.14819.2)
Supplement: Supplementary file 3 [file f1000research-7-17614-s0002.tgz › b2a79496-4ada-47c4-9682-a99ce867836d.zip!/Eight Iranian Health Journals/Journal of Knowledge and Health_43.docx]

© Journal of Knowledge and Health. This work is licensed under CC BY 4.0 (https://creativecommons.org/licenses/by/4.0/).

Volume & Issue: دوره 2، شماره 2: 1386

Title: نسبت های فعاليت آنزيم آرژيناز به فاکتورهای بيوشيميایی سرم بيماران همودياليزي قبل و بعد از دياليز به عنوان شاخص های بالينی جديد

http://knh.shmu.ac.ir/index.php/site/article/view/228

مقدمه: يافتن شاخص‌های پيش آگهي دهنده مي تواند در جهت تشخيص سريع­تر و تصميم‌گيری بهتر در روند درمان بيماران همودياليزی، پزشك را ياری دهد. در اين پژوهش، به عنوان يک پيشنهاد جديد، نسبت­های فعاليت آنزيم آرژيناز سرمی به ساير فاکتورهای بيوشيميایی سرم بيماران همودياليزي قبل و بعد از دياليز و افراد سالم بررسی و مقايسه گرديده و در نهايت به­­عنوان شاخص­های بالينی جديد در تشخيص ميزان پيشرفت بيماری و همچنين بررسی روند درمان ‌اندازه­گيری و مقايسه گرديده است. مواد و روش­ها: اين پژوهش بر روي 286 نمونه سرم خون انسان انجام شد. 206 نمونه سرم متعلق به بيماران همودياليزي (103 نمونه قبل و 103 نمونه بعد از دياليز) و 80 نمونه متعلق به افراد سالم بود. فعاليت آرژيناز به روش اوره، ازت اوره خون به روش اوره­آز، اسيد­اوريک به روش اصلاح شده فولين، کراتينين به روش ژافه و پروتئين تام به روش بيوره ‌اندازه‌گيری شد. نتایج: فعاليت آرژيناز سرم بين بيماران همودياليزي قبل از دياليز و بعد از دياليز با افراد سالم تفاوت معني‌داري نشان داد (01/0P<). همچنين فعاليت اين آنزيم بين بيماران همودياليزي قبل و بعد از دياليز تفاوت معني­داري نشان داد (01/0P<). به اين معني‌ كه عمل دياليز در كاهش فعاليت آنزيم آرژيناز مؤثر نبوده است. نسبت فعاليت آرژيناز سرمی به ازت اوره­ی خون، کراتينين و اسيد­اوريک سرم بين بيماران همودياليزي قبل از دياليز با افراد سالم و همچنين اين نسبت­ها بين بيماران همودياليزي قبل و بعد از دياليز تفاوت معني­داري نشان داد (01/0P<). به دليل اختلاف معنی‌داری که بين ميزان پروتئين تام سرم هر دو گروه سالم و نمونه­های بعد از دياليز، با نمونه­های قبل از دياليز وجود داشت، بين فعاليت ويژه‌ی آنزيم آرژيناز (نسبت فعاليت آرژينار به پروتئين تام) گروه‌های سالم، قبل و بعد از دياليز تفاوت معنی‌دار مشاهده نشد (01/0P<). نتیجه گیری: اين پژوهش پيشنهاد می­نمايد که نسبت­های فعاليت آرژيناز سرم بيماران همودياليزي به ساير فاکتورهای بيوشيميایی سرم به غير از پروتئين تام می‌تواند به عنوان شاخص‌های پيش آگهي دهنده در روند بهبود و درمان بيماران همودياليزي به كار گرفته شود.

Title: بررسی شیوع و خصوصیات درد¬گلو پس از بیهوشی عمومی از طریق لوله داخل تراشه در بیماران تحت عمل جراحی

http://knh.shmu.ac.ir/index.php/site/article/view/229

مقدمه: لوله‌گذاری داخل تراشه، تكنیكی استاندارد برای انجام بیهوشی عمومی است كه می‌تواند استرس زیادی برای بیمار ایجاد نماید این پژوهش به منظور بررسی شیوع و خصوصیات درد گلو پس از بیهوشی عمومی از طریق لوله داخل تراشه در بیماران تحت عمل جراحی باز شكستگی‌های ‌‌اندام‌های انتهایی انجام شده است. مواد و روش‌ها: در پژوهش توصیفی مقطعی حاضر، تعداد 78 بیمار 65-15 ساله تحت عمل جراحی باز شكستگی‌های ‌‌اندام‌ها به صورت غیرتصادفی و بر اساس مشخصات مورد نظر پژوهش‌گر انتخاب گردیدند. سپس با استفاده از پرسش‌نامه استاندارد درد مک‌گیل (فرم كوتاه شده) (McGill Pain Questionnaire-Short Form: (MPQ-SF)) وضعیت حسی، عاطفی، شدت درد و همچنین ارزیابی كلی درد گلو در هر یك از نمونه‌ها در طی دو فاصله زمانی یك ساعت و 24 ساعت پس از بیهوشی عمومی و خارج كردن لوله‌تراشه مورد سنجش قرار­گرفت. داده­ها با استفاده از آزمون‌های آماری توصیفی و استنباطی (تی مستقل، تی زوج، آنالیز واریانس یك‌طرفه و ضریب همبستگی پیرسون) مورد تجزیه و تحلیل و مقایسه قرار­گرفت. تجزیه و تحلیل یافته‌ها توسط نرم‌افزارSPSS  تحت ویندوز انجام شد. نتایج: بر اساس یافته‌های پژوهش، میانگین سنی واحدهای مورد پژوهش 29 سال بود و با توجه به نتایج حاصل از پرسش‌نامه استاندارد خلاصه شده درد مگ‌گیل، میانگین شاخص حسی درد گلو، یك و 24 ساعت پس از بیهوشی به ترتیب 42/3 و 2/1 و میانگین شاخص عاطفی درد­گلو، یك و 24 ساعت پس از بیهوشی به ترتیب 14/1 و 46/0 محاسبه گردید. هم‌چنین میانگین شدت درد­گلو بر اساس مقیاس بصری درد، یك و 24 ساعت پس از بیهوشی به ترتیب 51/3 و 18/1 برآورد­شد. 6/75% واحدهای پژوهش از درد­گلو در یك ‌ساعت پس از بیهوشی شكایت داشتند كه 24 ساعت پس از بیهوشی این میزان به 5/29% رسید. در كل اكثریت واحدهای مورد پژوهش (3/33%) یك‌ ساعت پس از بیهوشی شدت درد را ناراحت‌كننده ارزیابی نموده بودند. آزمون آماری تی زوج، تفاوت معنی‌دار بین شاخص حسی و عاطفی و هم‌چنین شدت درد گلو در یك و 24 ساعت پس از بیهوشی نشان داد. میانگین شدت درد گلو در زنان نسبت به مردان بیشتر گزارش­گردید. آزمون آماری ضریب همبستگی پیرسون ارتباط معنی‏دار و معكوسی بین شدت درد گلو و سن در 24 ساعت پس از بیهوشی نشان داد. نتیجه­گیری: با توجه به نتایج حاصل از پژوهش حاضر، هوشیاری نسبت به عوارض ناشی از بیهوشی عمومی از طریق لوله‌تراشه و بكارگیری راه‏كارهای مناسب جهت پیش گیری از عوارض پس از بیهوشی عمومی و افزایش رضایتمندی آنان از عمل جراحی یك ضرورت اساسی محسوب می‌گردد.

Title: بررسی میزان آگاهی کارکنان اتاق عمل در ارتباط با منابع احتراق و راه‌های پیش¬گیری از آن در اتاق ‏عمل

http://knh.shmu.ac.ir/index.php/site/article/view/231

مقدمه: احتراق یكی از مهم‌ترین حوادث ترسناك و وحشت‏آور برای بیمار و اعضای تیم جراحی است كه ممكن است در حین عمل جراحی اتفاق بیفتد. این پژوهش به منظور بررسی میزان آگاهی کارکنان اتاق عمل در ارتباط با منابع احتراق و راه‌های پیش­گیری از آن در اتاق ‏عمل انجام شده است. مواد و روش‌ها: در پژوهش توصیفی حاضر، نمونه‏گیری به صورت تمام شماری بوده و نمونه‏های پژوهش شامل كلیه کارکنان  تخصصی شاغل در اتاق عمل‌های بیمارستان‌های امام حسین (ع) و فاطمیه شاهرود (تعداد 48 نفر) بودند. در این پژوهش، میزان آگاهی نمونه ها از منابع احتراق         (منابع گرمایی، منابع اكسیژن و منابع قابل اشتعال) و راه‌های پیش­گیری از بروز حریق در اتاق عمل با استفاده از پرسش‌نامه‌ای طراحی شده در ارتباط با پیش­گیری و ایمنی از حریق در اتاق عمل مورد سنجش قرار گرفت. میانگین نمره آگاهی واحدهای مورد پژوهش در زمینه‌های یاد شده در بالا، بر مبنای 100-0 به سه سطح ضعیف، متوسط و خوب تقسیم‌بندی شد. داده­ها با استفاده از آزمون‌های آماری توصیفی و استنباطی (تی­مستقل، آنالیز واریانس یك‌طرفه و ضریب همبستگی پیرسون) مورد تجزیه و تحلیل و مقایسه قرار گرفت. نتایج: بر اساس نتایج حاصل از پژوهش، بیش­ترین درصد (9/97%) واحدهای مورد پژوهش، برگزاری برنامه آموزشی در ارتباط با پیش­گیری و ایمنی از حریق در اتاق عمل را ضروری عنوان نموده بودند و همگی (100%) این برنامه‌ها را با  اهمیت و سودمند می‌دانستند. اغلب (4/85%) واحدهای پژوهش اظهار نموده بودند كه تا به حال دوره آموزشی خاصی در ارتباط با پیش­گیری و ایمنی از حریق در اتاق عمل طی نکرده‌اند. میانگین آگاهی کارکنان اتاق عمل در ارتباط با احتراق و پیش­گیری از آن در متخصصین بیهوشی 7/15­%، تكنسین‌های اتاق عمل 1/13%، تكنسین‌های هوشبری 4/12% و در بین جراحان 9/10% برآورد گردید. دركل 4/60% از کارکنان اتاق عمل دارای سطح آگاهی متوسط، 3/33% ضعیف و 3/6% خوب در ارتباط با احتراق و پیش­گیری از آن در اتاق ‏عمل بودند. نتیجه­گیری: نتایج پژوهش حاضر نشان داد، متأسفانه اكثر کارکنان اتاق عمل از منابع احتراق و راه‌های پیش­گیری از آن آگاهی كافی ندارند. لذا بكارگیری شیوه‏های آموزشی مختلف جهت افزایش آگاهی کارکنان اتاق عمل در ارتباط با پیش­گیری و ایمنی از حریق در اتاق عمل پیشنهاد می‌گردد.

Title: طراحی مدل تمرین هوازی در آب و بررسی نقش آن در بهبود افسردگی

http://knh.shmu.ac.ir/index.php/site/article/view/233

مقدمه: افسردگي حالتي است كه بر كيفيت خلق­وخوي شخص تأثير عميق گذاشته و نحوه ادراك وي را از خويش و محيط اطرافش دگرگون  مي­سازد. اين بيماري فرد فعال را به موجودي با احساس بيهودگي، رانده شده از خود، فاقد اعتماد به نفس، خسته، گريزان از مردم و گاه پرخاشگر مبدل مي سازد كه همواره ناكام و ناراضي است. اگر چه  این اختلال عمدتاً بوسیله دارو مورد درمان قرار گرفته است تحقیقات گسترده در چند سال اخیرحاکی است که تمرینات استقامتی می‌توانند نقش عمده ای در بهبود اختلالات مختلف خلقی ایجاد نمایند. همچنین نتایج کلینیکی و نوروبیولوژیک اخیر پیشنهاد نموده‌اند که تمرینات جسمانی حتی می‌توانند به عنوان یک روش درمانی جایگزین در مورد برخی از اختلالات خلقی مورد استفاده قرار گیرند. در این پژوهش آثار  الگوی تمرینی طراحی شده در دانشجویان با اختلال افسردگی مورد بررسی قرار گرفت. مواد و روش­ها: در این پژوهش 249نفر دانشجوی پسر بصورت تصادفی در آزمون اولیه شرکت نمودند و آزمون بک جهت ‌اندازه گیری میزان افسردگی آنان مورد استفاده قرار گرفت. 52 نفر از نمونه­ها که نمره افسردگی بیش از 18 را کسب نمودند جهت شرکت در این مطالعه انتخاب شدند. برنامه تمرینی شامل 60 دقیقه بازی یا توپ در آب (شبیه واتر پلو) بود که 3جلسه در هفته تکرار و به مدت 7 هفته ادامه می یافت. شدت تمرین در هر جلسه حدود 60 تا 70 در صد ضربان قلب بیشینه بود. آزمون بک در چهار مرحله از شرکت کنندگان بعمل آمد. آزمون اول دو ماه قبل از شروع تمرین، آزمون دوم روز اول تمرین، آزمون سوم در هفته چهارم تمرین، و آزمون چهارم در هفته هفتم تمرین به انجام رسید. نتایج: میانگین نمره بک نمونه‌ها در روز قبل از شروع تمرین  8/8±  19/25،  در هفته چهارم  بعد از تمرین به 8/8 ± 08/15 و در هفته هفتم تمرین به  2/8 ± 64/11 رسیده که کاهش معنی‌دار نسبت به روز اول تمرین با 05/0P<  نشان داد.  نتیجه­گیری:  از نتايج فوق چنين به نظر مي رسد كه الگوی تمرين هوازي طراحی شده در آب مي­تواند به عنوان يك روش درمانی موثر مورد توجه قرارگرفته و در صورت انجام پژوهش­های تکمیلی حتی به عنوان یک روش درمان جایگزین در برخی از بیماران مبتلا به افسردگی تحت نظارت پزشک مربوطه بکار رود.

Title: بررسي ميزان آگاهي، نگرش و مهارت رابطين بهداشتي شهر شاهرود در مورد خودآزمايي پستان

http://knh.shmu.ac.ir/index.php/site/article/view/234

مقدمه: سرطان پستان شايع‌ترين سرطان زنان و دومين علت مرگ­و­مير ناشي از سرطان در بين زنان است. ميزان مرگ­ومير اين سرطان به‌طور مستقيم در ارتباط با مرحله بيماري در زمان تشخيص است و در صورت استفاده از روش‏هاي غربالگري، كاهش مي‌يابد. يكي از مهم­ترين راه­هاي تشخيص‌ زودرس سرطان پستان، خودآزمايي پستان است. اين مطالعه با هدف تعيين ميزان آگاهي، نگرش و مهارت رابطين بهداشتي در زمينه خودآزمايي پستان انجام شده است. مواد و روش­ها: 89 نفر از رابطين بهداشتي 20 تا 57 ساله شاهرود به روش تصادفي انتخاب و آگاهي، نگرش و مهارت آنان درمورد خودآزمايي پستان با استفاده از پرسش‌نامه سنجيده شد. اطلاعات حاصله با استفاده از روش‏هاي آماري تجزیه و تحلیل گرديد. نتايج: 63% رابطين بهداشتي در مواد و روشهای تشخیص زودرس سرطان پستان، علایم و نشانه­ها، اطلاعاتی داشتند. آگاهي آنان از روش صحيح انجام خودآزمايي پستان درحد متوسط بود. 51% رابطين، خودآزمايي را در زمان مناسب انجام مي‌دادند، 78% رابطين نسبت به خودآزمايي پستان نگرش مثبت داشتند. بين نگرش و تأهل ارتباط آماري معني‌دار ديده شد. 4/12% از مهارت خوبي برخوردار نبودند. نتیجه­گیری: در مجموع با توجه به آگاهي متوسط و مهارت ضعيف رابطين بهداشتي لزوم برگزاري دوره‌هاي آموزشي خاص توصيه مي‌شود. از طرف ديگر با انجام مطالعات بيش­تر بايد عواملي را كه باعث تشويق زنان به استفاده از روش‏هاي غربالگري سرطان پستان می گردند را شناسایي نمود.

Title: بررسی آگاهی و نگرش زنان باردار مراجعه‌کننده به مراکز بهداشتی درمانی شهر قم نسبت به روش‌های ختم بارداری

http://knh.shmu.ac.ir/index.php/site/article/view/237

مقدمه: زایمان طبیعی بهترین روش زایمانی در اکثریت زنان باردار محسوب می‌شود و بر اساس جدیدترین آمارها در کشور (سال 1382) 33% از زایمان‌ها به روش سزارین انجام می‌گیرد. فقدان آگاهی از عوارض سزارین و نگرش منفی نسبت به زایمان طبیعی، یکی از مهم‌ترین علل افزایش تمایل زنان باردار نسبت به سزارین می‌باشد. هدف از این مطالعه، بررسی آگاهی نگرش زنان باردار نسبت به روش‌های ختم بارداری می‌باشد. مواد و روش‌ها: در این مطالعه مقطعی، 136 مادر باردار به روش تمام­شماری که بصورت متوالی به هشت مرکز بهداشتی درمانی شهر قم مراجعه می‌کردند مورد بررسی قرار گرفتند. جهت جمع‌آوری اطلاعات پرسش‌نامه­ای شامل خصوصیات دموگرافیک، عبارات آگاهی سنجی و نگرش سنجی نسبت به زایمان طبیعی و سزارین که با نظرخواهی از ماماها و متخصصان زنان و زایمان تهیه و پس از تأیید پایایی آن با روش بازآزمایی، از طریق مصاحبه با بیمار تکمیل گردید. جهت تجزیه و تحلیل داده‌ها از آزمون‌های2 c و آزمون فیشر استفاده­ شد. نتایج: سطح آگاهی مادران باردار در خصوص روش‌های ختم بارداری در 4/40% موارد متوسط و در 6/59% موارد خوب بود. نگرش مادران نسبت به زایمان واژینال در 1/94% و نسبت به سزارین در 2/63% موارد مثبت بود. 9/5% مادران نسبت به زایمان طبیعی و 36% نسبت به سرازین بی­نظر بودند. بین نگرش مادران نسبت به سزارین با سطح آگاهی آن‌ها ارتباط آماری معنی‌دار مشاهده شد (0001/0P<)، اما بین نگرش نسبت به زایمان طبیعی با سطح آگاهی مادران ارتباطی دیده نشد (19/0=P). نتیجه گیری: سطح آگاهی و نگرش نسبت به روش‌های ختم بارداری نسبت به سال های قبل بهبود یافته است، با توجه به معنی­دار بودن ارتباط بین آگاهی و نگرش نسبت به سزارین، با برنامه‌ریزی صحیح جهت افزایش آگاهی و ارتقاء نگرش مثبت نسبت به روش‌های ختم بارداری می‌توان میزان تمایل به سزارین را در مادران باردار کاهش داد.

Title: ارزيابي فرم کوتاه شده پرسش‌نامه درد مك‌گيل در زنان باردار نخست‌زاي مراجعه¬كننده به زايشگاه فاطميه شاهرود

http://knh.shmu.ac.ir/index.php/site/article/view/238

مقدمه: پرسش­­نامه درد مک گيل ابزار مناسب و  قابل اعتمادی  برای درمان انواع دردهای مزمن و حاد با شدت متوسط تا شدید می­باشد و  به زبان­های مختلفی ترجمه و تاييد شده است. هر چند طی سال­های اخیر استفاده از فرم کوتاه شده پرسش­نامه درد مك گيل توصيه شده است، اما  پرسش­نامه مذکور تنها به دو زبان فرانسه و انگلیسی، ترجمه و مورد استفاده قرار­گرفته­است. مطالعه حاضر به ارزيابی پرسش­نامه کوتاه شده درد مك­گيل در زنان باردار مراجعه­کننده به بيمارستان فاطميه شاهرود می­پردازد. مواد و روش‌ها: تعداد 140 زن باردار نخست­زا با حاملگی تک­قلو و نمايش سر که با دردهای خود­به­خودی به زايشگاه فاطميه شاهرود مراجعه­می­کردند در اين مطالعه قرار­گرفتند. هيچ­يك از واحد­هاي مورد مطالعه روش­هاي دارویي­ و­ غير­دارویي تسكين درد را دريافت نمی­كردند. شدت درد در مرحله اول زايمان با استفاده از معيار کلامی عددی (10-0 نمره) و در مرحله دوم زايمان با استفاده از فرم پرسش­نامه کوتاه شده درد مک­گيل تعيين شد. پرسش­نامه شامل 11 و 4 جزء به­ترتيب مربوط به اجزاي حسي و عاطفي درد، هم چنين معيار كلامي درجه­بندي شده شدت درد ( 10-0 نمره) و  انتخاب يكي از 6 معيار شدت درد (­بدون درد تا درد شكنجه آور) بود كه با پرسش از واحدهاي مورد مطالعه در مرحله دوم درد تكميل شده است. یافته‌ها: در اين مطالعه ميانگين نمره شدت درد بر اساس فرم اصلاح شده درد مك­گيل 9/35 (از مجموع 59 نمره) بود. هيچ يك از واحدهاي مورد پژوهش درد ضرباني نداشتند و بيش­ترين مورد مربوط به بيان درد شديد از اجزاي حسي و ابراز ترس شديد از زايمان، از اجزاي عاطفي درد به ترتيب در 90 و 3/44 درصد از واحد هاي مورد پژوهش بود. 42 درصد از واحد­هاي مورد پژوهش درد زايمان را در مرحله دوم زايمان، شكنجه آور بيان نمودند. نتیجه­گیری:  نظر به اينكه در اين مطالعه هيچ يك از واحد­هاي مورد پژوهش شكايت از درد ضرباني نداشتند و با توجه به اهميت بررسي اعتبار فرم كوتاه شده درد مك­گيل، بررسي بيشتر بر روي وجود اين جزء حسي درد در پرسش­نامه مذکور با بررسي اجزاي حسي درد در جمعيت بيشتري از زنان باردار پيشنهاد مي­گردد.

Title: بررسي اپیدمیولوژیک موارد خودآزاری عمدی (مسمومیت) در شهرستان شاهـرود

http://knh.shmu.ac.ir/index.php/site/article/view/240

مقدمه: خودکشی اصلی‌ترین اورژانس روانپزشکی و یکی از بزرگترین معضلات بشری است، لذا مطالعه حاضر به بررسی خصوصیات اقدام­کنندگان به خودآزاری عمدی (مسمومیت­ها)، مواد و داروهایی که مورد استفاده آن‌ها قرار گرفته و برخی عوامل همراه با آن می پردازد. مواد و روش‌ها: این مطالعه مقطعی بر روی 383 بیمار اقدام­کننده به خود آزاری عمدی از نوع مسمومیت مراجعه کننده به بیمارستان           امام حسین (ع) شاهرود از تیر ماه سال 81 تا پایان خرداد سال 82 انجام شد. داده­ها از طریق پرسش‌نامه­های طراحی شده و انجام مصاحبه با بیمار و بستگان او گرد­آوری و با استفاده از نرم‌افزار SPSS مورد تجزیه و تحلیل قرار گرفت. یافته­ها: در طول یک سال، تعداد 383 نفر (163 نفر مرد و 220 نفر زن) مورد بررسی قرار گرفتند که گروه سنی 30-15 سال بیشترین فراوانی را (4/80%) به خود اختصاص دادند. 9/61% آن‌ها مجرد و اکثریت آن‌ها (8/48%) تحصیلات زیر دیپلم داشته و 5/88% موارد اقدام به خود آزاری را شهرنشین­ها انجام داده بودند، بیشترین موارد اقدام به خود آزاری، با استفاده از داروهای شیمیایی (341) نفر انجام شده که بنزودیازپینها (26%) شایعترین گروه دارویی مورد استفاده بود. نتیجه‌‌‌گیری: ­نتایج مطالعه حاضر نشان داد که اقدام به خودکشی در گروه جوانان و مجردین قابل توجه بوده و اقدامات حفاظتی می‌تواند در اولویت اول، به بررسی مشکلات این گروه­ها اختصاص ­یابد. همچنین از مهم‌ترین مباحث بالینی، توجه به عوامل خطر خودکشی و درمان موارد  خود­آزاری عمدی جهت جلوگیری از تکرار آن می‌باشد.
